# HMOs Impact the Gut Microbiome of Children and Adults Starting from Low Predicted Daily Doses

**DOI:** 10.3390/metabo14040239

**Published:** 2024-04-20

**Authors:** Danica Bajic, Frank Wiens, Eva Wintergerst, Stef Deyaert, Aurélien Baudot, Pieter Van den Abbeele

**Affiliations:** 1 DSM Nutritional Products Ltd., Wurmisweg 576, 4303 Kaiseraugst, Switzerland; 2 Cryptobiotix SA, Technologiepark-Zwijnaarde 82, 9052 Ghent, Belgium

**Keywords:** short-chain fatty acids, human milk oligosaccharides, microbial metabolite, intestinal microbiota, 2′FL, LNnT, 3′SL, 6′SL, LC-MS, shotgun sequencing

## Abstract

Recent studies suggest that the dietary intake of human milk oligosaccharides (HMOs) provides health benefits from infancy up to adulthood. Thus far, beneficial changes in the adult gut microbiome have been observed at oral doses of 5–20 g/day of HMOs. Efficacy of lower doses has rarely been tested. We assessed four HMO molecular species—2′Fucosyllactose (2′FL), Lacto-N-neotetraose (LNnT), 3′Sialyllactose (3′SL), and 6′Sialyllactose (6′SL)—at predicted doses from 0.3 to 5 g/day for 6-year-old children and adults (*n* = 6 each), using ex vivo SIFR^®^ technology (Cryptobiotix, Ghent, Belgium). This technology employing bioreactor fermentation on fecal samples enables us to investigate microbial fermentation products that are intractable in vivo given their rapid absorption/consumption in the human gut. We found that HMOs significantly increased short-chain fatty acids (SCFAs), acetate, propionate (in children/adults), and butyrate (in adults) from predicted doses of 0.3–0.5 g/day onwards, with stronger effects as dosing increased. The fermentation of 6′SL had the greatest effect on propionate, LNnT most strongly increased butyrate, and 2′FL and 3′SL most strongly increased acetate. An untargeted metabolomic analysis revealed that HMOs enhanced immune-related metabolites beyond SCFAs, such as aromatic lactic acids (indole-3-lactic acid/3-phenyllactic acid) and 2-hydroxyisocaproic acid, as well as gut–brain-axis-related metabolites (γ-aminobutyric acid/3-hydroxybutyric acid/acetylcholine) and vitamins. The effects of low doses of HMOs potentially originate from the highly specific stimulation of keystone species belonging to, for example, the *Bifidobacteriaceae* family, which had already significantly increased at doses of only 0.5 g/day LNnT (adults) and 1 g/day 2′FL (children/adults).

## 1. Introduction

The gut microbiome is involved in diverse physiological pathways critical to human health, including the fermentation of undigested dietary carbohydrates in the colon, a process which results in the release of short-chain fatty acids (SCFAs) [1,2]. SCFAs, especially acetate, propionate, and butyrate, modulate important signaling pathways in the gut and the immune homeostasis of the human host [2,3,4,5]. Due to the more frequent application of metabolomics in research on human nutrition and health, new bioactive molecules at the gut microbiome–host interface are being discovered [6,7]. For example, *Bifidobacterium* spp., which is associated with various health benefits [2,8,9,10,11], were recently shown to metabolize aromatic amino acids into aromatic lactic acids (e.g., indole-3-lactic acid from tryptophan), which are released into the intestinal lumen where they support immune development in early life [12].

A specific example of carbohydrates that stimulate the microbial production of SCFAs and aromatic lactic acids by enhancing *Bifidobacterium* spp. are human milk oligosaccharides (HMOs) [13]. HMOs are the third most abundant solid component of breast milk [14]: the top 12 most abundant HMOs in breast milk include fucosylated HMOs, such as 2′-fucosyllactose (2′-FL), neutral HMOs, like lacto-N-neotetraose (LNnT), and sialylated structures, including 3′Sialyllactose (3′SL) and 6′Sialyllactose (6′SL) [15]. A recent ex vivo study demonstrated the potential of HMOs to exert health benefits beyond infancy [13]; when supplied to the microbiome of children and adults, 2′FL/LNnT potently stimulated *Bifidobacterium* spp. In contrast, traditional prebiotics (inulin and fructo-oligosaccharides (FOS)) were only bifidogenic for adults. A study by Elison et al. (2016) tested doses of 5, 10, or 20 g/day of 2′FL and/or LNnT consumed over the course of 2 weeks for their effects on gut microbiota in healthy adults [16]. They found a clear bifidogenic effect that increased with HMO doses (at the expense of lower average fractional abundances of Proteobacteria and Firmicutes). We hypothesized that even doses lower than 5 g/day might be effective for inducing beneficial changes in the microbiomes of adults and children.

Clinical trials are essential to demonstrate health benefits, but due to large intra- and inter-individual variability [17], they are less suited for deciphering subtle yet relevant effects on gut microbes. In contrast, preclinical in vitro/ex vivo studies minimize variability and enable insights into metabolites that are intractable in vivo due to fast absorption from the gut lumen [18,19]. Nevertheless, translation from preclinical to clinical results is often a hurdle [20]. A key limitation is that in vivo derived microbiota often significantly change when transferred to a lab environment, both in short-term [21,22,23] and long-term gut models [24,25]. Moreover, the current generation of gut models suffers from the issue of low throughput, reducing the number of controls and/or replicates. Biological replicates are critical for addressing interpersonal differences that largely exceed variations along colonic regions as well as differences between the lumen and mucus [17]; they are also known to impact treatment outcomes [26]. The recently developed SIFR^®^ technology (Systemic Intestinal Fermentation Research) is a high-throughput, miniaturized, and bioreactor-based technology in which the in vivo derived microbiota of multiple parallel study subjects are cultivated in presence of specific treatments. A key feature is the sustained similarity between the in vivo derived microbiota and microbiota in SIFR^®^ reactors [27]. This technology has been shown to produce insights down to a microbial species level (within only 24–48 h), which is predictive of clinical effects [27]. A final important aspect of SIFR^®^ technology is the translatability of ex vivo effective doses into oral daily in vivo doses.

After recently assessing the impact of four single HMOs (2′FL, LNnT, 3′SL and 6′SL) compared to traditional prebiotics (inulin and FOS) at a dose equivalent to 5 g/day [13], the key objective of the current study was to identify their impact on metabolite production and microbial composition in children and adults, upon treatment with lower doses of these four single HMOs. This research question was addressed by testing doses ranging from human equivalent doses of 0.3 to 5 g/day and using SIFR^®^ technology combined with a state-of-the art multi-omics approach (quantitative shotgun sequencing and untargeted metabolomics).

## 2. Materials and Methods

### 2.1. Test Compounds

**Figure 1:**
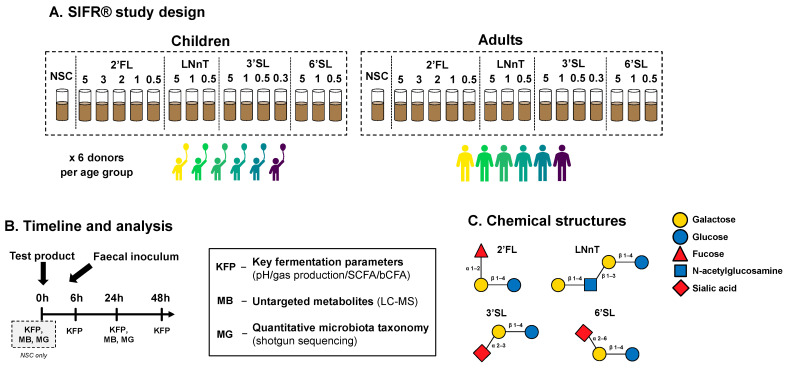
**Experimental design to assess dose-dependent effects of HMOs on the gut microbiota of children and adults.** (**A**) Reactor design using ex vivo SIFR^®^ technology to assess how four single HMOs (2′FL, LNnT, 3′SL, 6′SL), with doses ranging from equivalents of 0.3 to 5 g/day, impact the gut microbiota of 6-year-old children and human adults, compared to an untreated NSC (*n* = 6 per age group). (**B**) Timeline and analysis at different time points. (**C**) Chemical structure of HMOs.

Four HMOs were investigated: 2′Fucosyllactos (2′FL—GlyCare^TM^ 2FL 9000—batch #: 20156002), Lacto-N-neotetraose (LNnT—GlyCare^TM^ LNnT 9000—batch #: 20135001), 3′Sialyllactose (3′SL—GlyCare^TM^ 3SL 9001—batch #: 19421101) and 6′Sialyllactose (6′SL –GlyCare^TM^ 6SL 9001—batch #: 19487101) (Figure 1C). These test products were provided as powders by DSM Nutritional Products Ltd. (Kaiseraugst, Switzerland). A no-substrate control (NSC) was also included, in which the microbial inoculum was grown in the absence of additional test products.

### 2.2. SIFR^®^ Technology

SIFR^®^ technology was recently validated and enables the study of the human gut microbiome in a highly biorelevant manner across numerous parallel test conditions (both treatments and test subjects) [27]. Briefly, individual bioreactors were processed in parallel in a bioreactor management device (Cryptobiotix, Ghent, Belgium). Each reactor contained 5 mL of a nutritional medium-fecal inoculum blend supplemented with between 0.3 and 5 g test compound/L, which was sealed individually, before being rendered anaerobic. Blend M0003 (Cryptobiotix, Ghent, Belgium) was used for the preparation of a nutritional medium. Subsequently, bioreactors were incubated under continuous agitation (140 rpm) at 37 °C for 48 h (MaxQ 6000, Thermo Scientific, Thermo Fisher Scientific, Merelbeke, Belgium). Upon gas pressure measurement, liquid samples were collected for subsequent analysis.

Fresh fecal samples were collected according to a procedure approved by the Ethics Committee of the University Hospital Ghent (reference number BC-09977). This involved the participant (or their parents) signing an informed consent in which they donated their (or their child’s) fecal sample for the study. The selection criteria for human adults were as follows: no antibiotic use in the past 3 months, no gastrointestinal disorders (cancer, ulcers, IBD), no use of probiotics, no smoking, alcohol consumption < 3 units/d and 20 < BMI < 25. Four male and two female adults with an average age of 30 (±4 years) participated in the study. Furthermore, three male and three female 6-year-old children were included (6 ± 0 years). Stool consistency, measured by the Bristol Stool Score (BSS), was used to rule out abnormal stool pointing in dysbiotic gut microbiota [28]. BSS was within the normal range for all subjects and similar between both age groups (BSS adults = 3.33 ± 0.52; BSS children 3.33 ± 1.03). Hence, we ruled out lifestyle, health conditions, and stool consistency as confounders in our analysis of age as a modulator of the microbiome response to HMO supplementation.

### 2.3. Experimental Design, Timeline, and Analysis

A kinetic study was performed, simulating the colonic fermentation of HMOs by the gut microbiota in healthy 6-year-old children or adults (*n* = 6 per age group) (Figure 1A,B). The study design consisted of an untreated no-substrate control (NSC) and four HMOs tested at a range of doses (equivalent to 0.3 up to 5 g/day) in a single technical replicate across four time points (0, 6, 24 and 48 h), each of which was performed in an independent reactor. The test doses were chosen according to a rationale based on previous ex vivo studies, existing clinical evidence of effective doses, and regulatory recommendations. Firstly, the equivalent of 5 g/day was chosen as the highest test dose, since such a dose was likely to produce significant effects based on previous studies of HMOs tested at this dose, and resulted in clinically relevant findings [27]. Furthermore, EFSA recommends doses lower than 5 g/day for several specified HMOs. Maximal use levels of 3 g/day are recommended for 2′FL [29], while the daily doses of 3′SL should not exceed 0.5 g/day for children and adults [30]. Moreover, the recommended maximal use levels for LNnT and 6′SL are 1.8 g/day [31] and 1.5 g/day [29], respectively. Therefore, we decided to test all HMOs at doses equivalent to 1 g/day and 0.5 g/day. In addition, we tested 3′SL at 0.3 g/day.

Key fermentation parameters (0, 6, 24, and 48 h) and microbial composition were examined and an in-depth analysis of metabolite production via untargeted metabolomics was conducted (both at 0 h for the NSC, and at 24 h for all test arms).

### 2.4. Key Fermentation Parameters

SCFAs (acetate, propionate, butyrate, and valerate) and branched-chain fatty acids (bCFA; sum of isobutyrate, isocaproate, and isovalerate) were determined via gas chromatography with flame ionization detection, upon diethyl ether extraction, as previously described [32]. Briefly, 0.5 mL samples were diluted in distilled water (1:3), acidified with 0.5 mL of 48% sulfuric acid, after which an excess of sodium chloride was added along with 0.2 mL of internal standard (2-methylhexanoic acid) and 2 mL of diethyl ether. Upon homogenization and separation of the water and diethyl ether layer, diethyl ether extracts were collected and analyzed using a Trace 1300 chromatograph (Thermo Fisher Scientific, Merelbeke, Belgium) equipped with a Stabilwax-DA capillary GC column, a flame ionization detector, and a split injector using nitrogen gas as the carrier and makeup gas. The injection volume was 1 µL and the temperature profile was set from 110 °C to 240 °C. The carrier gas was nitrogen, and the temperatures of the injector and detector were 240 and 250 °C, respectively. pH was measured using an electrode (Hannah Instruments Edge HI2002, Temse, Belgium).

### 2.5. Microbiota Phylogenetic Analysis: Quantitative Shotgun Sequencing

Initially, a bacterial cell pellet was obtained by centrifugation (5 min at 9000× *g*) of 1 mL liquid sample collected at 0 h or 24 h from the reactors. DNA was extracted via the SPINeasy DNA Kit for Soil (MP Biomedicals, Eschwege, Germany), according to the manufacturer’s instructions. Subsequently, DNA libraries were prepared using the Nextera XT DNA Library Preparation Kit (Illumina, San Diego, CA, USA) and IDT Unique Dual Indexes with total DNA input of 1 ng. Genomic DNA was fragmented using a proportional amount of Illumina Nextera XT fragmentation enzyme. Unique dual indexes were added to each sample, followed by 12 cycles of PCR to construct libraries. DNA libraries were purified using AMpure magnetic Beads (Beckman Coulter, Brea, CA, USA), eluted in QIAGEN EB buffer, quantified using a Qubit 4 fluorometer and a Qubit dsDNA HS Assay Kit, and sequenced on an Illumina Nextseq 2000 platform 2 × 150 bp. Unassembled sequencing reads were converted to relative abundances (%) using the CosmosID-HUB Microbiome Platform (CosmosID Inc., Germantown, MD, USA; https://app.cosmosid.com/; accessed on 17 January 2022) [33,34]. This platform was also used to calculate the Chao1 and Simson diversity index.

Next, quantitative data were obtained by correcting relative abundances (%) with total cell counts for each sample (cells/mL; flow cytometry), resulting in estimated cell counts/mL of different taxonomic groups. For total cell count analysis, liquid samples were diluted in anaerobic phosphate-buffered saline (PBS), after which cells were stained with SYTO 16 at a final concentration of 1 µM and counted via a BD FACS Verse flow cytometer (BD, Erembodegem, Belgium). Data were analyzed using FlowJo, version 10.8.1.

### 2.6. Untargeted Metabolomics Analysis

Liquid chromatography–mass spectrometry (LC-MS) analysis was carried out on a Thermo Scientific Vanquish LC coupled to Thermo Q Exactive HF MS (Thermo Scientific), using an electrospray ionization source. The analysis was performed both in negative and positive ionization mode. Ultra-performance liquid chromatography was performed by applying a slightly modified version of the protocol described by Doneanu et al. [35]. Peak areas were extracted using Compound Discoverer 3.1 (Thermo Scientific), along with a manual extraction based on an in-house library using Skyline 21.1 (MacCoss Lab Software) [36].

Compounds were identified at different levels, i.e., level 1—retention times (compared against in-house authentic standards), accurate mass (with an accepted deviation of 3 ppm), and MS/MS spectra; level 2a—retention times and accurate mass; level 2b—accurate mass and MS/MS spectra; and level 3—accurate mass alone. A total of 2027 compounds were detected: 410 were annotated on level 3, 62 on level 2b, 62 on level 2a, and 43 on level 1. Technical variability was tested by running a QC sample (pooled sample of all samples) every six samples. These QC samples were grouped together in an exploratory analysis (level 1–annotated metabolites), confirming the high reproducibility of the method (Figure S1). This high reproducibility suggests that any variation observed is truly due to a treatment and not due to technical variation.

### 2.7. Data Analysis and Bioinformatics

For the exploratory evaluation of the obtained results, a series of principle component analyses (PCA) was performed using GraphPad Prism (v9.3.1; www.graphpad.com; accessed on 17 January 2022). The same software was used to make a series of boxplots, bar charts, and heat maps. While boxplots and bar charts present actual values, heat maps present log_2_-transformed fold changes for the different treatments compared to the parallel control arm (NSC). This way, when a metabolite or microbial group is increased by a given treatment, a positive value is displayed, while negative values reveal a decrease. For the statistical analysis of treatment effects, differences between study arms were compared with the NSC using a repeated-measures ANOVA analysis (based on paired testing), and *p*-values were corrected with Benjamini–Hochberg (FDR = 0.05 or 0.10 as specified). Paired testing was performed to ensure that the microbiota of the same 6 test subjects was considered across all test arms.

For the analysis of microbial composition, three measures were taken, as elaborated by Van den Abbeele et al. (2023) [27]. Firstly, the statistical analysis was performed on log_10_-transformed values. Secondly, a value of a given taxonomic group below the limit of detection (LOD) was considered equal to the overall LOD. Finally, a threshold was set to retain the 100 most abundant species in the analysis to avoid excessive *p*-value corrections.

Furthermore, regularized canonical correlation analysis (rCCA) was performed to highlight correlations between metabolites (key fermentation parameters and metabolomics data) and compositional data (at species level). Regarding compositional data, log-transformed, absolute phylogenetic data were used as inputs. rCCA was executed using the mixOmics package with the shrinkage method for estimation of penalization parameters (version 6.16.3) in R (4.1.1; www.r-project.org; accessed on 26 December 2022) [37].

Finally, differences in baseline microbiota composition between children and adults were assessed via a PCoA based on Bray–Curtis distance along with linear discriminant analysis effect size (LEfSe) at a species level, using the CosmosID-HUB Microbiome. This enabled the identification of the taxa most likely to explain differences between children and adults (LDA threshold = 2).

## 3. Results

### 3.1. First Findings: (i) Age-Dependent Gut Microbiome Composition between Children and Adults, and (ii) Kinetic Sampling Covered Saccharolytic and Proteolytic Fermentation

Children and adult donors had a significantly different fecal microbiota (*p* = 0.024 based on Bray–Curtis distance) (Figure S2A), with *Bifidobacteriaceae* being a key differentiator: while the children’s microbiota was enriched with *B. catenulatum* and *B. pseudocatenulatum*, the adults’ microbiota was enriched with *B. adolescentis* (Figure S2B). This provides a rationale for assessing the dose-dependent effects of HMOs separately for both age groups.

**Figure 2:**
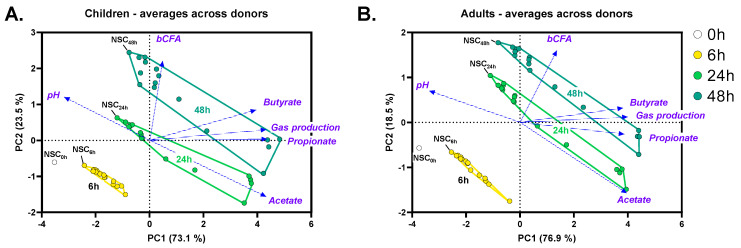
**Kinetic sampling covered saccharolytic (0–24 h) and proteolytic fermentation processes (24–48 h).** Principal component analysis (PCA) summarizing the levels of key fermentation parameters (pH, SCFA, bCFA and gas production), as averaged for six children (**A**) or six adults (**B**) at different time points (0, 6, 24, and 48 h) in the no-substrate control (NSC) or upon treatment with four HMOs (2′FL, LNnT, 3′SL, 6′SL) at doses ranging from equivalent of 0.3 to 5 g/day.

The gut microbiota of children and adults was metabolically active throughout the entire 48 h incubation period, as illustrated by the differential clustering of 0, 6, 24, and 48 h samples in PCA plots based on key fermentation parameters (Figure 2A,B). The kinetic sampling covered profound saccharolytic fermentation (0–24 h) and any subsequent proteolytic fermentation (bCFA production; 24–48 h). One could thus compare the observations in the aforementioned time frames with the observations along the proximal (0–24 h) and distal colon (24–48 h). Given the focus on the effect of HMOs (carbohydrates), the 24 h time point was selected for in-depth analysis of dose-dependent effects of HMOs on microbial composition and metabolite production.

### 3.2. From the Lowest Doses Onwards (0.3–0.5 g/d), HMOs Significantly Impacted Key Fermentation Parameters

**Figure 3:**
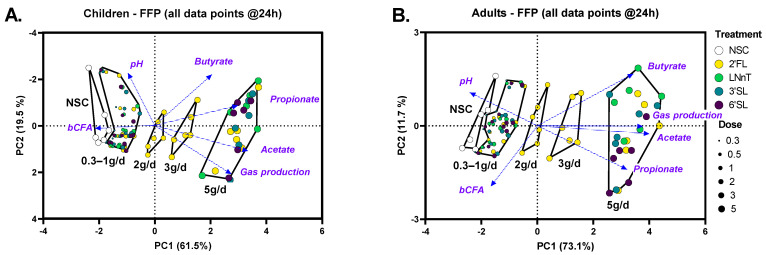
**HMOs exerted dose-dependent effects on key fermentation parameters from the lowest test doses onwards.** A principal component analysis (PCA) summarizing the levels of fundamental fermentation parameters (pH, SCFA, bCFA and gas production), as averaged across 6 children (**A**) or adults (**B**) at different time points (0, 6, 24, and 48 h) in the no-substrate control (NSC) and upon treatment with HMOs (2′FL, LNnT, 3′SL, 6′SL) at doses ranging from equivalents of 0.3 to 5 g/day.

All four HMOs (2′FL, LNnT, 3′SL, 6′SL) impacted key fermentation parameters at 24 h in a dose-dependent manner, with HMO-treated samples being distinguishable from the NSC from the lowest doses of 0.3–0.5 g/day onwards, both for children and adults (Figure 3A,B). Despite interpersonal differences, observable from the spread of samples within each treatment cluster, treatment effects were generally consistent for the six children and adults (for optimal visualization, individual subjects (rather than treatments) are highlighted in Figure S3).

**Figure 4:**
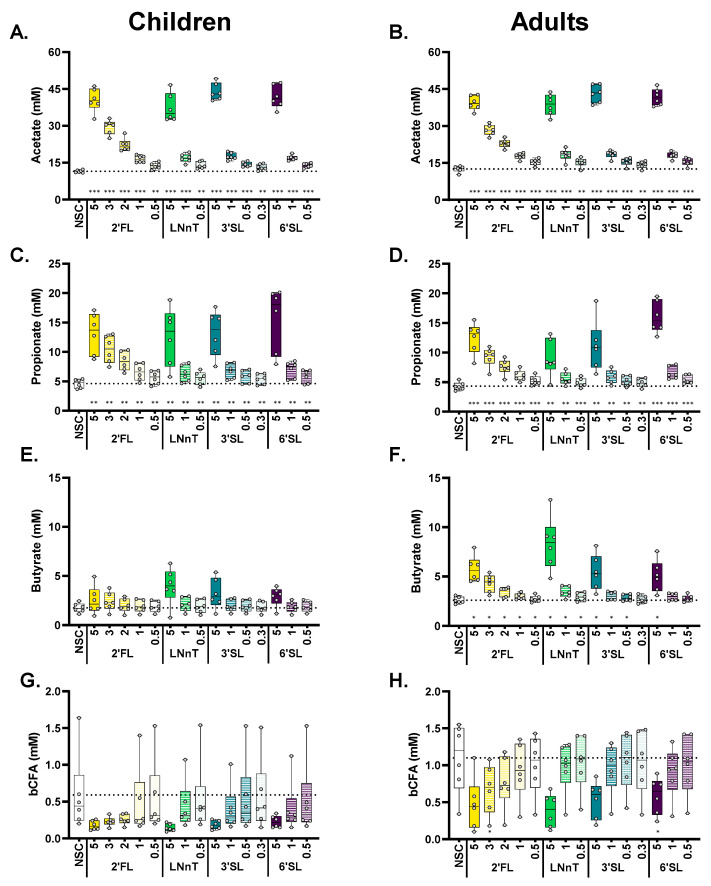
**When administered to the children’s microbiota, the four HMOs significantly increased acetate/propionate from the lowest test dose onwards. For the adult microbiota, all HMOs also significantly increased butyrate (with few exceptions).** The impact of HMOs (2′FL, LNnT, 3′SL, 6′SL) at doses ranging from equivalents of 0.3 to 5 g/day on acetate (**A**,**B**), propionate (**C**,**D**), butyrate (**E**,**F**), and bCFA (**G**,**H**) levels in simulated gut microbiota of children (**A**,**C**,**E**,**G**; *n* = 6) or adults (**B**,**D**,**F**,**H**; *n =* 6), at 24 h upon initiation of treatment, compared to a no substrate control (NSC), as tested with the ex vivo SIFR^®^ technology. Statistical differences between treatments and NSC are indicated with asterisks [* (*p*_adjusted_ < 0.05), ** (*p*_adjusted_ < 0.01) or *** (*p*_adjusted_ < 0.001)]. The dashed horizontal line indicates the average value of each parameter in the NSC. bCFA = branched fatty acids.

Statistical analysis revealed that each HMO, from its lowest test dose onwards, significantly increased acetate (Figure 4A,B), propionate (Figure 4C,D), and total SCFA (Figure S4E,F), while significantly decreasing pH (Figure S4A,B), both for children and adults. 6′SL exerted a strong effect on propionate production for adults at 5 g/day (Figure 3D), while for children, larger inter-individual differences in propionate levels were noted (Figure 4C). Effects on butyrate were more pronounced for adults compared to children (Figure 4E,F), with all test products (particularly LNnT) significantly increasing butyrate for adults, except for the low doses of 3′SL (0.3 g/day) and 6′SL (0.5/1 g/day). bCFA levels markedly decreased for all treatments at 5 g/day, both for children and adults, yet, given the large inter-individual differences in the NSC, only tendencies were noted (Figure 4G,H). Finally, gas production also increased in a dose-dependent manner (Figure S4C,D) with changes being significant with only a few exceptions (lowest dose of 3′SL (children) and 6′SL (children/adults)). Notably, 6′SL resulted in minor gas production for adults compared to the other HMOs, despite the marked increase in SCFA production.

### 3.3. HMOs Increased Bacterial Cell Density, While Generally Maintaining High Diversity

**Figure 5:**
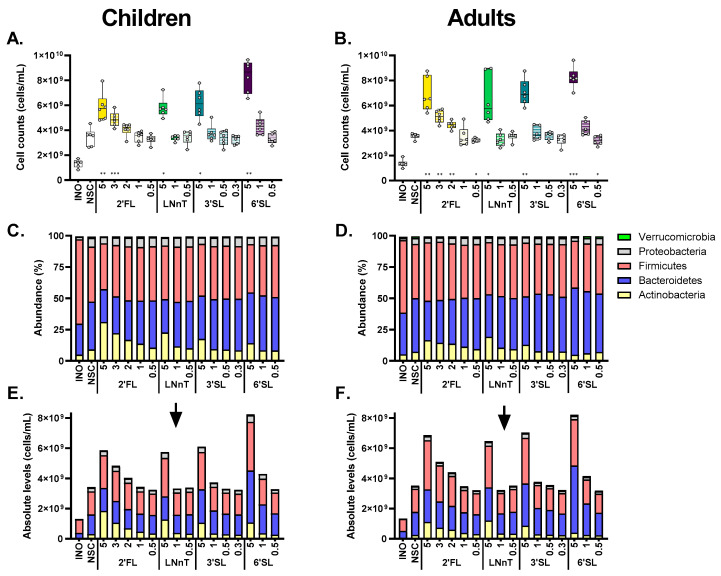
**All HMOs tended to increase bacterial cell density, reaching significance from 3 g/day onwards, thus stressing the need to convert sequencing data from proportional (%) to quantitative values (cells/mL).** The impact of four HMOs (2′FL, LNnT, 3′SL, 6′SL) at doses ranging from equivalents of 0.3 to 5 g/day on cell density (**A**,**B**), and microbial composition at the phylum level, as averaged over simulations for 6-year-old children and adults (*n* = 6), presented as proportional (%) (**C**,**D**) and absolute values (cells/mL) (**E**,**F**) compared to a no-substrate control (NSC), as tested with the ex vivo SIFR^®^ technology. Samples were collected after 0 h (INO) and after 24 h of simulated colonic incubations. Statistical differences between treatments and NSC are indicated with asterisks [* (*p*_adjusted_ < 0.05), ** (*p*_adjusted_ < 0.01) or *** (*p*_adjusted_ < 0.001)].

There was a marked increase in bacterial cell density between the 0 h (INO) and 24 h samples of the untreated control (NSC), by a factor of 2.7 ± 0.6, both for children and adults (Figure 5A,B). At the same time, microbial diversity remained high (Figure S5A–D), illustrating that the increased cell density between 0–24 h was due to the growth of a broad spectrum of gut microbes, thus confirming the effective operation of the ex vivo SIFR^®^ model.

At the highest dose (5 g/day), all HMOs significantly increased cell density compared to the NSC (Figure 5A,B). At lower doses, there was a trend towards increased cell density that was significant for 2′FL from 3 g/day onwards. The Chao1 diversity index (reflecting species richness) was similar for all treatments and the untreated control for adults, while for children, a significant decrease was observed for a limited number of treatments (mostly high doses of 2′FL and 3′SL), suggesting that specialist species became highly abundant, which was confirmed by lower values of the reciprocal Simpson diversity index (that accounts for species richness and evenness) at higher doses (Figure S5A–D).

Given the differences in cell numbers across samples, we corrected proportional data obtained via sequencing with total cell numbers obtained from flow cytometry to gain insights into the true changes in microbial composition. This conversion from proportional data (%; Figure 5C,D) to absolute data (cells/mL; Figure 5E,F) is visualized at phylum level, as averaged across the six test subjects per age group.

### 3.4. HMOs Exerted Strong Effects on Bifidobacteriaceae and/or Bacteroidaceae at Low Test Doses

**Figure 6:**
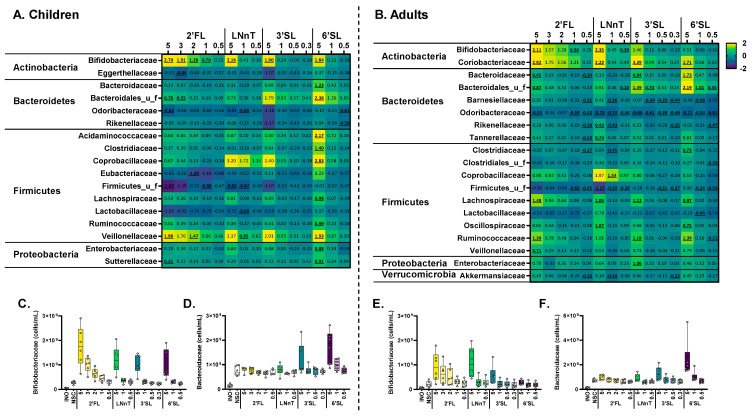
**All HMOs exerted bifidogenic effects in children, with 2′FL/LNnT additionally exerting bifidogenic effects for adults.** Sialylated HMOs (3′SL/6′SL) specifically increased *Bacteroidaceae*. The impact of four HMOs (2′FL, LNnT, 3′SL, 6′SL) at doses ranging from equivalents of 0.3 to 5 g/day on microbial composition at a family level at 24 h upon initiation of treatment, as tested with the ex vivo SIFR^®^ technology for (**A**) children (*n* = 6) or (**B**) adults (*n* = 6). The heatmaps represent average values of microbial taxa that were significantly affected by any of the treatments (FDR = 0.10). Significant differences are indicated by the bold and underline of the average log2 (abundance treatment/abundance NSC). (**C**–**F**) Violin plots representing the abundances (cells/mL) of *Bifidobacteriaceae* and *Bacteroidaceae* for children (**C**,**D**) and adults (**E**,**F**). Samples represented in the violin plots were collected at 0 h (INO = inoculum) and after 24 h of incubation.

The impact of HMOs on microbial composition was first analyzed at a family level (Figure 6). For children, all HMOs markedly increased *Bifidobacteriaceae*, which was most pronounced for 2′FL and already significant from 1 g/day onwards (Figure 6A,C). Also for adults, 2′FL and LNnT markedly increased *Bifidobacteriaceae,* with this increase being significant for treatments with as low as 1 g 2′FL/d and 0.5 g LNnT/d (Figure 6A,E). Furthermore, 6′SL, and to a lesser extent 3′SL, boosted *Bacteroidaceae* (Figure 6D,F). Especially for adults, there was a remarkable difference in *Bacteroidaceae* stimulation between these two sialylated HMOs (that only differ in sialic acid positioning). This elevated *Bacteroidaceae* abundance likely increased the abundance of the succinate converting *Phascolarctobacterium faecium* (part of the *Acidaminococcaceae*), which most significantly increased upon 6′SL treatment in children. Another example of potential cross-feeding was noted with Veillonellaceae, which likely consumed lactate produced by Bifidobacteriaceae. Finally, a series of Firmicutes families, including Coprobacillaceae, Lachnospiraceae, Oscillospiraceae, and Ruminococcaceae, displayed HMO-specific and dose-dependent increases.

Dose-dependent effects were confirmed at a species level, both for children (Figure S6) and adults (Figure S7). Moreover, this additional analysis at the lowest taxonomic level demonstrated that, compared to children, there were distinct species involved in HMO fermentation for adults. For example, the potent bifidogenic effect of HMOs was due to their stimulatory effects on five different *Bifidobacterium* spp. for children (Figure S6A), in contrast to the two *Bifidobacterium* spp. for adults (Figure S7A). Further, while 6′SL stimulated *Bacteroides fragilis* for children, 6′SL also increased *Phocaeicola dorei, Phocaeicola vulgatus,* and *Phocaeicola massiliensis* for adults.

Moreover, by performing an rCCA analysis between SCFA data and microbial composition at a species level (for significantly/consistently affected species), correlations could be established between species that were responsible for increased SCFA production upon HMO treatment (Figures S6C–F and S7C–F). For example, upon the treatment of adult microbiota with 2′FL, acetate production was likely driven by *Bifidobacterium adolescentis*, propionate production by *Mediterraneibacter faecis/Ruminococcus torques*, and butyrate production by *Anaerobutyricum hallii* (Figure S7C). In contrast, in treatment with 6′SL, acetate/propionate production was likely driven by *Bacteroides*_u_s/*Ruminococcus*_u_s/*Oliverpabstia intestinalis*, propionate by *Phocaeicola vulgatus,* and butyrate by *Gemmiger formicilis* (Figure S7F). Thus, substrate-specific consortia specifically fermented the provided HMOs.

### 3.5. HMOs Impacted a Range of Health-Related Metabolites beyond SCFA at Low Test Doses

**Figure 7:**
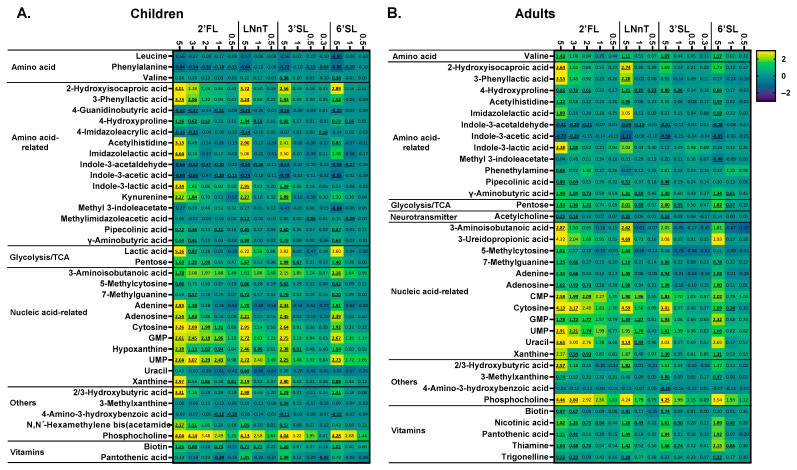
**HMOs impacted a range of health-related metabolites, well beyond SCFA, from low doses onwards.** Heat maps showing the impact of four HMOs (2′FL, LNnT, 3′SL, 6′SL) at doses ranging from equivalents of 0.3 to 5 g/day on a selection of metabolites (annotated at level 1, 2a, 2b or 3 and previously linked with the human gut microbiota) as quantified via untargeted LC-MS after 24 h of incubation, as tested using SIFR^®^ technology for (**A**) children (*n* = 6) or (**B**) adults (*n* = 6). The reported metabolites were significantly affected by any of the treatments (FDR = 0.20). Significant differences are indicated by the bold and underline of the average log2 (abundance treatment/abundance NSC).

The untargeted LC-MS metabolomics analysis provided comprehensive insights into the metabolic output of microbial communities. In line with the potent modulation of SCFA production by HMOs, a marked stimulation of a series of human-health-related metabolites was observed. Similar to SCFA production, there was a marked dose–response effect with the impact on metabolite production being more profound as test doses increased up to 5 g/d. Moreover, at the highest test dose, after 24 h of treatment, there was a striking difference between 3′SL/6′SL compared to LNnT/2′FL, particularly for adults (Figure 7).

Aromatic lactic acids (indole-3-lactic acid and 3-phenyllactic acid) were strongly affected by HMOs. For adults, 2′FL/LNnT were the strongest modulators, while 3′SL/6′SL also stimulated aromatic lactic acids for children. For 2′FL, significant effects were observed from 3 g/day onwards for indole-3-lactic acid (adults) and 3-phenyllactic acid (children).

Particularly, 2′FL/LNnT stimulated 2-hydroxyisocaproic acid (HICA) (for 2′FL, from 3 g/day on), with 3′SL/6′SL again specifically increasing HICA in children.

Potent effects were observed on metabolites related to the gut–brain axis, i.e., γ-aminobutyric acid (GABA), hydroxybutyric acid (HBA), and acetylcholine. Both for children and adults, GABA increased for all HMOs at 5 g/day (for 2′FL even for 3 g/d). For adults, GABA production was most pronounced and significantly increased from 1 g/day LNnT/3′SL/6′SL and 2 g/day 2′FL. HBA significantly increased at the highest test dose for 2′FL/LNnT (both age groups) and 3′SL/6′SL (children only), with acetylcholine significantly increasing for adults (3–5 g/day 2′FL, 5 g/day LNnT, and 5 g/day 3′SL).

For both age groups, all HMOs at 5 g/day (for 2′FL from 3 g/day on) also significantly increased 7-methylguanine levels. For adults, 2′FL/LNnT specifically increased β-aminoisobutyric acid (BAIBA) levels, while 2′FL/6′SL increased BAIBA for children. For both age groups, all HMOs (already from doses of 1–2 g/day onwards for 2′FL and LNnT) boosted a series of ribonucleotides including GMP/UMP (for children also CMP). Finally, in terms of B vitamins, significantly increased levels of four B vitamins were noted upon HMO treatment for adults. The impact on pantothenic acid/thiamine (vitamin B5/B1) was of interest given the production of HMOs during the 48 h incubation. Pantothenic acid significantly increased for all HMOs (5 g/d), but especially for 3′SL and 6′SL. Thiamine most markedly increased for 6′SL. Interestingly, low doses of HMOs significantly increased thiamine levels (2 g/day 2′FL, 1 g/day 3′SL, 1 g/day 6′SL).

To link the production of these metabolites to specific microbial species, an rCCA was performed between compositional data (significantly/consistently affected species) and significantly affected metabolites, for each individual product, both for children (Figure S8) and adults (Figure S8). This revealed correlations between the presence of specific species and the production of specific metabolites.

## 4. Discussion

We evaluated how various low doses of four single HMOs, ranging from human equivalent doses of 0.3 to 5 g/day impacted the gut microbiota of children and adults. The adopted ex vivo SIFR^®^ technology is uniquely designed to predict dose effects on gut microbiota modulation down to species level in clinical studies [27]. The increase in *Bifidobacteriaceae* and *A. hallii* caused by 2′FL and LNnT for adults in this study, for example, is in line with published clinical data [16,38]. Another feature of this technology is the minimal bias between the original donor microbiota and the microbiota growing in SIFR^®^ reactors, which was critical for studying age-related differences in the gut microbiome. Again mirroring in vivo findings [39,40], *B. pseudocatenulatum* and *B. catenulatum* were abundant in children donors, whereas *B. adolescentis* was abundant in adult donors. Such differences greatly impact outcomes of interventions [13]. Furthermore, SIFR^®^ technology also circumvents issues that render clinical studies ill-suited for unravelling gut microbiota modulation, such as the inability to sample at the site of fermentation, the rapid absorption of microbial metabolites in humans and overall large inherent variation in microbiota composition within a single individual over time. In contrast, ex vivo studies can be performed in a highly controlled manner, thus establishing cause–consequence relationships between, in casu, the additional administration of low HMO doses and changes in the gut microbiome of children and adults. The key finding of the study is that HMOs already significantly impact health-associated microbial taxa and related metabolites from doses that are well below those commonly applied in clinical studies involving adults (5–20 g/day) [16,38]. Moreover, the current study provides a roadmap for a more rational selection of the four HMOs under investigation and their doses when setting up clinical studies that aim to improve health by targeting the human gut microbiome.

All four HMOs significantly enhanced SCFA production from 0.3 to 0.5 g/day onwards. Each dose of each HMO significantly increased acetate and propionate, both in children and adults, with each dose of each HMO (except the lowest dose of 3′SL/6′SL) also significantly increasing butyrate for adults. Such stimulatory effects of HMOs on SCFA production by human gut microbes have been observed before [13,27,41,42]. The multitude of health benefits reportedly associated with SCFA production [2,4,5] suggests that the consumption of HMOs could contribute to health benefits, already from doses of 0.3–0.5 g/day onwards. Human in vivo colonic butyrate levels (as apparent from fecal butyrate levels and the percentage of butyrate producing bacteria in fecal samples) increase along with the transition from an infant- to an adult-like gut microbiota. Reported age specific molar ratios of colonic butyrate are around 5% in infants [43,44] and 15–20% in adults [44,45]. Moreover, Derrien et al. (2019) revealed an enrichment of acetate/propionate-producing gut microbes in school children (e.g., various *Bifidobacterium, Bacteroides,* and *Prevotella* spp.) as opposed to butyrate-producing species in adults (*Anaerobutyricum hallii* and *Clostridium butyricum*). Our finding of lower responses in relative butyrate concentration to HMO substrates in samples from 6-year-old children vs. adult samples is in general agreement with these reports.

Moreover, HMO-specific effects were noted with LNnT, specifically an increase in butyrate, while 6′SL most markedly increased propionate (especially for adults), again in line with earlier observations [13]. For children, the increase in butyrate was linked to the presence of butyrate, producing *Anaerobutyricum hallii,* a species that has recently been shown to cross-feed with *Bifidobacterium* spp. to produce butyrate, among other metabolites [46]. The observation of such well-characterized, HMO-dependent effects at low doses could help to design future clinical trials. When the aim is, for instance, to achieve health benefits by upregulating the production of butyrate (the main energy source of colonocytes exerting health benefits mostly within the gut [2,47]), LNnT is the recommended HMO, with a dose of only 1 g/day exerting potent effects compared to 2 g/day 2′FL (adults). On the other hand, when the aim is to upregulate the gut production of propionate, which enters the bloodstream and exerts health benefits beyond the gut [48,49], 6′SL is the preferred HMO, with a propionate production at 1 g/day, exceeding propionate levels observed for any of the other HMOs at this dose.

Quantitative sequencing enabled accurate insights into gut microbial composition as it removes the noise that would otherwise be introduced in proportional sequencing outcomes (%), when test products impact cell density (such as HMOs that increase cell density) [27]. An initial key finding was that all HMOs markedly increased *Bifidobacteriaceae* for children, with 2′FL and LNnT also markedly increasing *Bifidobacteriaceae* for adults. *Bifidobacterium* spp. are indeed specialized in HMO fermentation [50,51]. This bifidogenic effect also explains the potent increases in acetate for these study arms as *Bifidobacterium* spp. degrade HMOs to acetate and lactate through the bifid shunt, a dedicated pathway involving phosphoketolase activity [52]. *Bifidobacteriaceae* have been linked to health benefits from infancy [8,11,12,53] up to adulthood [40], which has led to the development of *B. longum* and *B. adolescentis* strains as probiotics [54]. When a clinical study would aim to achieve health benefits in adults by enhancing *Bifidobacteriaceae* levels, 2′FL or LNnT are thus promising substrates given the similar or more potent effects of these substrates when dosed at only 1 g/day, even compared to 5 g/day 6′SL. In contrast, 6′SL specifically increased other health-related species such as *Phocaeicola dorei* for adults [55,56] and *Bacteroides fragilis* for children [57,58,59]. Only 1 g/day 6′SL exerted similar or more potent effects on these microbial taxa compared to 2′FL at 5-fold higher doses. The specific stimulation of *P. dorei* is in line with recent findings that *P. dorei* isolates strongly fermented HMOs containing sialic acid (N-acetylneuraminic acid), while being unable to ferment neutral and fucosylated HMOs [60]. Furthermore, *B. fragilis* has been shown to possess a particular sialidase that enables this pioneering species to colonize [61]. In the succinate pathway [62,63], these species were likely key species involved in the production of high levels of propionate for 6′SL. Overall, the significant effects of low doses of HMOs could thus originate from the highly specific stimulation of aforementioned keystone species. Such high specificity might result in less substrate being used by collateral, non-beneficial gut microbes. HMOs could thus potentially be classified as high-specificity prebiotics unlike traditional prebiotics [64,65].

The untargeted metabolomics analysis elucidated the health-promoting potential of HMOs beyond the stimulation of SCFA production. The lowest HMO doses (0.3 g/d), both for children and adults, already induced measurable increases in specific microbial metabolites. The various test products enhanced the production of aromatic lactic acids such as indole-3-lactic acid and 3-phenyllactic acid, which are shown to be produced by *Bifidobacterium* spp. from the aromatic lactic acids, tryptophan and phenylalanine, respectively [12]. These aromatic lactic acids are linked to immune function [12] and brain processes via the aryl hydrocarbon receptor [66,67,68,69]. Furthermore, different HMOs also stimulated 2-hydroxyisocaproic acid (HICA, a leucine derivative shown to be produced by lactic acid bacteria, with antimicrobial [70,71] and anti-inflammatory activity [72]), and gut–brain-axis-related metabolites (γ-aminobutyric acid (GABA, [73]: formed via the decarboxylation of glutamate [74] by *Bifidobacterium* spp. [75]), 3-hydroxybutyric acid (3-HBA, [76]), and acetylcholine [77]). Moreover, stimulatory effects were noted in 3-aminoisobutanoic acid (BAIBA: protective in cardiometabolic disease [78]), CMP/GMP/UMP (building blocks of DNA/RNA present in breast milk [79], linked with the hypnotic action of breast milk [80]), vitamins (biotin, nicotinic acid, pantothenic acid and thiamine), hydroxyproline (antioxidant [81]), and hypoxanthine (considered protective in irritable bowel syndrome (IBS) [82]). As summarized in Table 1, in line with effects on SCFA production and microbial composition, significant HMO-dependent, dose-dependent, and age-dependent effects were thus noted for the broad panel of metabolites under investigation. For instance, in contrast to adult microbiota, 3′SL and 6′SL stimulated *Bifidobacterium*-driven metabolites, such as aromatic lactic acids and HICA, in line with an increase in a variety of *Bifidobacterium* species when 3′SL and 6′SL were dosed to the microbiota in children (particularly *B. pseudocatenulatum)*. This stresses the specificity by which HMOs stimulate specific members of the gut microbiome and how this elicits the production of specific metabolites that may confer specific health benefits.

Moreover, as many of these metabolites are not directly produced from HMOs but rather from specific amino acids, HMOs thus indirectly stimulate the production of these metabolites. A first explanation of this indirect effect could be the HMO-mediated enhanced growth of specific microbial species capable of converting amino acids to said metabolites (e.g., *Bifidobacterium* spp.). Another mechanism could be that HMOs also change the environmental conditions of the gut. For example, by lowering intraluminal pH, HMOs could enhance the production of GABA as this metabolite is indeed produced as an acid stress response [83].

This study has several limitations. Firstly, while the absence of a host component in SIFR^®^ technology provides insights into metabolite production and microbial composition that are hard to obtain in vivo, the absence of a host also implies that the findings of the current study should be considered as complementary to clinical studies, rather than as a replacement. Despite the high predictivity of SIFR^®^ technology for clinical findings [49], the findings of the current study are yet to be confirmed in future clinical trials. Furthermore, while preclinical research with gut models was, until recently, often only performed with a single test subject [84], the inclusion of six subjects per age group in the current study could be considered as a relatively low number, given the vast interpersonal differences among humans [17]. While six donors per age group was sufficient to unravel significant differences between children and adults in line with clinical findings [39,40], it could have been interesting to increase the number of test subjects to, for instance, twenty-four [32].

Overall, the key finding of the current ex vivo study is that HMOs already significantly impact the relative abundance of health-associated taxa in the microbial gut community and the production of host-health-related microbial metabolites (well beyond SCFA) from predicted doses that are well below those that have been applied in clinical studies thus far (5–20 g/day). The effects of such low doses of HMOs potentially originate from the highly specific stimulation of keystone species belonging to, among others, the *Bifidobacteriaceae* family that significantly increased at only 0.5 g/day LNnT (adults) and 1 g/day 2′FL (children/adults). The broad range of metabolites that were stimulated have been linked with immune health, and the gut–brain axis, while also having been shown to provide protection against cardiometabolic disease and IBS. It would be interesting to set up clinical studies to demonstrate potential health benefits of such low doses of HMOs. Besides dose, the type of HMO provided as a substrate for microbial fermentation had a clear influence on microbial composition and metabolite production. Overall, this study provides insights for the rational selection of HMO types and doses during future clinical studies that aim to improve the health of children or adults via specific modulation of the gut microbiota.

## Figures and Tables

**Table 1:** Untargeted metabolomics (LC-MS) revealed that HMOs impacted a range of health-related metabolites, well beyond SCFA, from low doses onwards. The table indicates which dose, ranging from 0.3 to 5 g/day, of which HMO (2′FL, LNnT, 3′SL, 6′SL) significantly enhanced a given metabolite when dosed to the children’s (indicated with ‘C’ and green shading) or adults’ (indicated with ‘A’ and yellow shading) microbiota, as tested using SIFR^®^ technology.

Relevance	Metabolite	2′FL (g/day)					LNnT (g/day)			3′SL (g/day)				6′SL (g/day)		
		5	3	2	1	0.5	5	1	0.5	5	1	0.5	0.3	5	1	0.5
Immune—Brain [12,66,67,68,69]	Indole-3-lactic acid	A/C	A				C			C				C		
	3-phenyllactic acid	A/C	C				A/C			C				C		
Immune—Antimicrobial [70,71,72]	HICA	A	A/C				A/C			C				C		
Brain [73,76,77]	GABA	A/C	A/C	A			A/C	A		A/C	A			A/C	A	
	3-HBA	A/C					A/C			C				C		
	Acetylcholine	A	A				A			A						
Cardiometabolic [78]	BAIBA	A/C					A							C		
Building blocks DNA/RNA [79]	CMP	A	A	A			A	A		A				A		
	GMP	A/C	A/C	A/C	C		A/C	A		A/C				A/C		
	UMP	A/C	A/C	A/C	C		C	A		A/C				A/C		
B vitamins	Biotin	A/C	A/C		C		A/C	C		A/C				C		
	Nicotinic acid	A	A	A	A		A	A		A	A			A	A	
	Pantothenic acid	A	A				A/C			A/C				A		
	Thiamine	A	A	A			A			A	A			A	A	
Antioxidant [81]	Hydroxyproline	A/C	A/C	A/C			A/C	A/C	A	A/C	A			A/C	C	
IBS [82]	Hypoxanthine	C	C	C	C		C	C		C	C			C		

## Data Availability

The datasets generated during and/or analyzed during the current study are available from the corresponding author upon reasonable request.

## Supplementary Materials

The following supporting information can be downloaded at: https://www.mdpi.com/article/10.3390/metabo14040239/s1. Figure S1: Significant metabolite production occurred between 0 and 24 h, while the QC samples tightly co-localized. Figure S2: Fecal microbiota composition of children (6 years old) and adults was fundamentally different. Figure S3: HMOs exerted dose-dependent effects on key fermentation parameters from the lowest test dose onwards. Figure S4: When administered to the microbiota of children and adults, the four HMOs decreased pH, while increasing gas production and total SCFA levels (significant with only few exceptions). Figure S5: When administered to children and adult microbiota, the four HMOs generally maintained a high microbial diversity. Figure S6: All HMOs impacted specific microbial species when supplied to the microbiota of children. Figure S7: All HMOs impacted specific microbial species when supplied to the adult microbiota. Figure S8: Correlation between metabolites and species after HMO treatment—children. Figure S9: Correlation between metabolites and species after HMO treatment—adults.
