# Supplementary material for: HMOs Impact the Gut Microbiome of Children and Adults Starting from Low Predicted Daily Doses
Source: Metabolites. 2024 Apr 20;14(4):239. doi: 10.3390/metabo14040239 (PMC11052010; doi:10.3390/metabo14040239)
Supplement: Supplementary file 1 [file metabolites-14-00239-s001.zip › metabolites-2918530-supplementary.pdf]

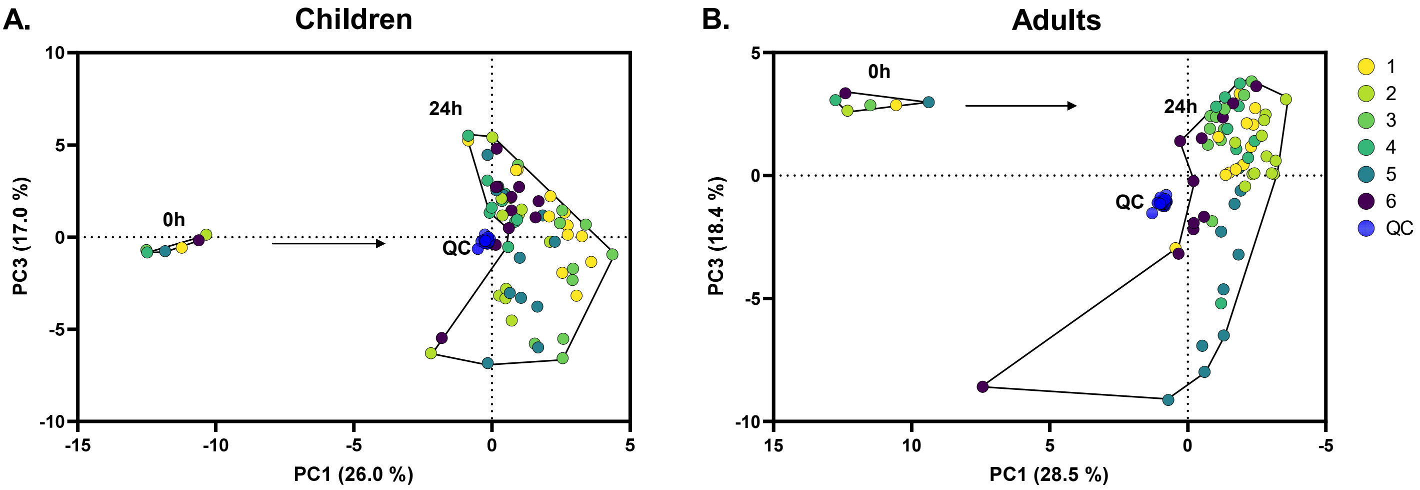

Figure S1. Marked metabolite production occurred between 0-24h, while the QC samples tightly co-localized. Principal component analysis (PCA) of level 1-annotated metabolites (LC-MS) for children (A) and adults (B) (n = 6), both at 0h and 24h of treatment with four HMOs (2′FL, LNnT, 3′SL, 6′SL) at doses ranging from equivalent of 0.3 to 5 g/day. QC = quality control sample (= pooled sample of all samples); LC-MS = Liquid chromatography coupled with mass spectrometry.

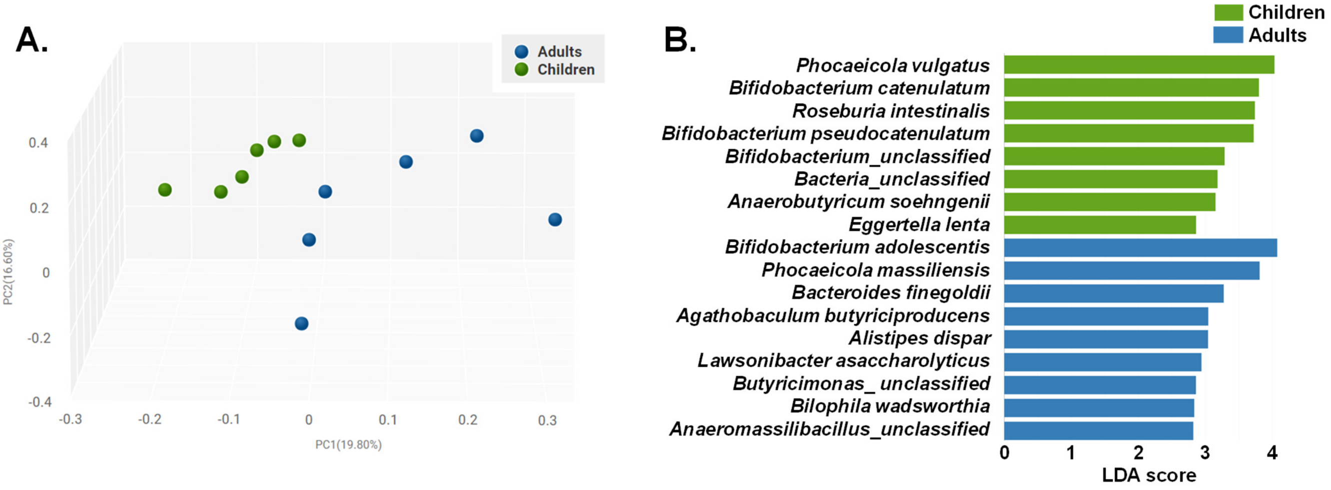

Figure S2. Fecal microbiota composition of children (6 years old) and adults was fundamentally different. (A) Principal coordinates analysis (PCoA) based on Bray-Curtis distance for microbial species (%), as quantified via shallow shotgun sequencing (p = 0.024). (B) Linear discriminant analysis effect size (LEfSe) at species level shows the taxa most likely to explain differences between children and adults (LDA threshold = 2).

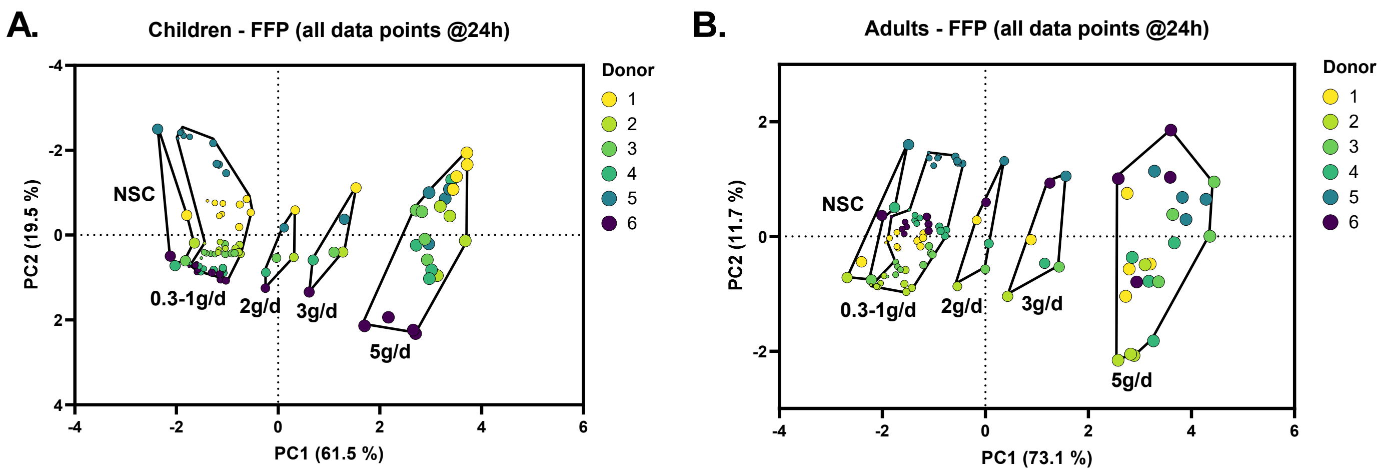

Figure S3. HMOs exerted dose-dependent effects on key fermentation parameters from the lowest test dose onwards. Principal component analysis (PCA) summarizing the levels of fundamental fermentation parameters (pH, SCFA, bCFA and gas production), as averaged across 6 children (A) or human adults (B) at different time points (0, 6, 24 and 48h) in the no substrate control (NSC) and upon treatment with four HMOs (2′FL, LNnT, 3′SL, 6′SL) at doses ranging from equivalent of 0.3 to 5 g/day. **Figure S3 is identical to Figure 2, yet with visualization of the test subjects rather than the treatments**.

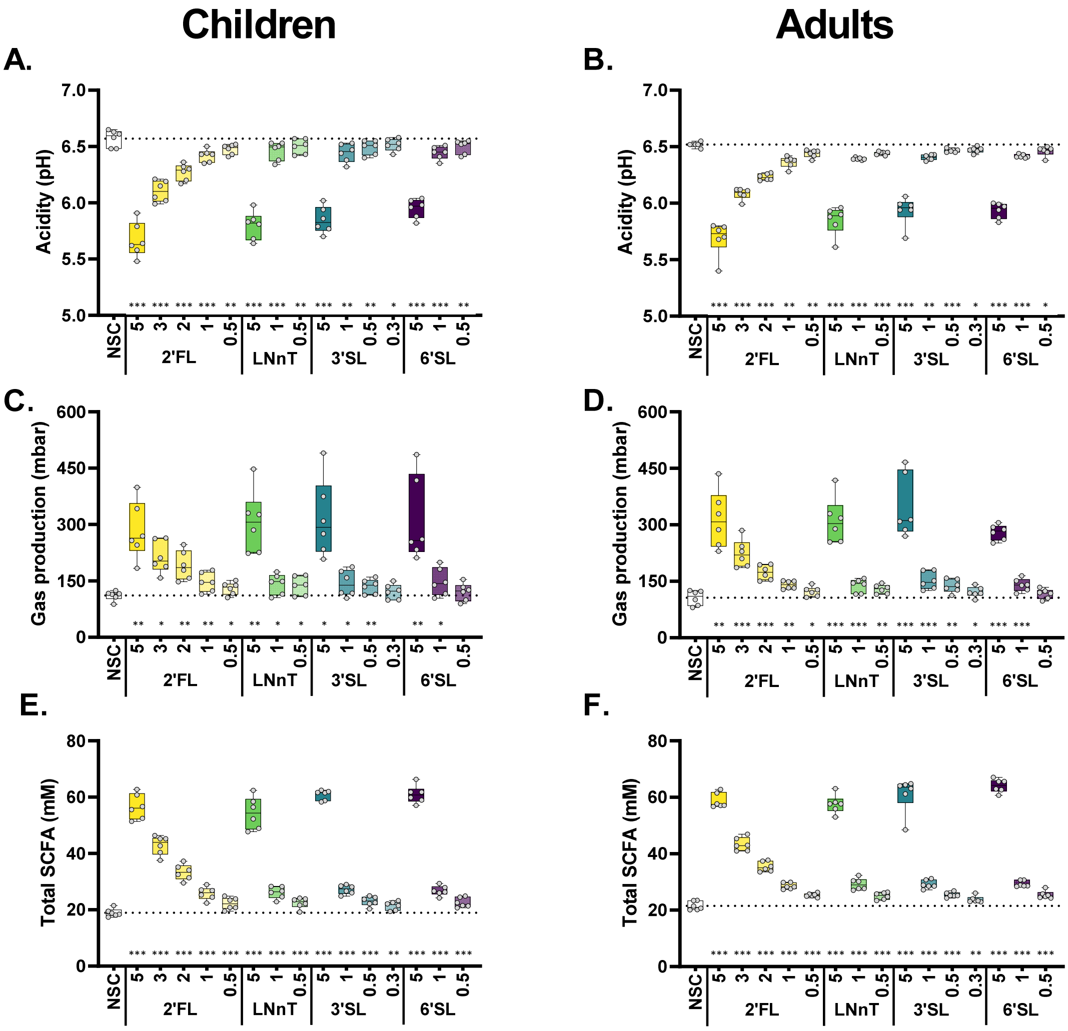

Figure S4. **When administered to the children's and adult microbiota, the four HMOs decreased pH, while increasing gas production and total SCFA levels (significant with only few exceptions).** Impact of four HMOs (2′FL, LNnT, 3′SL, 6′SL) at doses ranging from equivalent of 0.3 to 5 g/day on acetate (A, B), propionate (C, D), butyrate (E, F), and bCFA (G, H) levels for simulated gut microbiota of children (A, C, E, G; n = 6) or adults (B, D, F, H; n = 6), at 24h upon initiation of treatment, compared to a no substrate control (NSC), as tested with the *ex vivo* SIFR® technology. Statistical differences between treatments and NSC are indicated with asterisks [* ($p_{adjusted} < 0.05$), ** ($p_{adjusted} < 0.01$) or *** ($p_{adjusted} < 0.001$)].

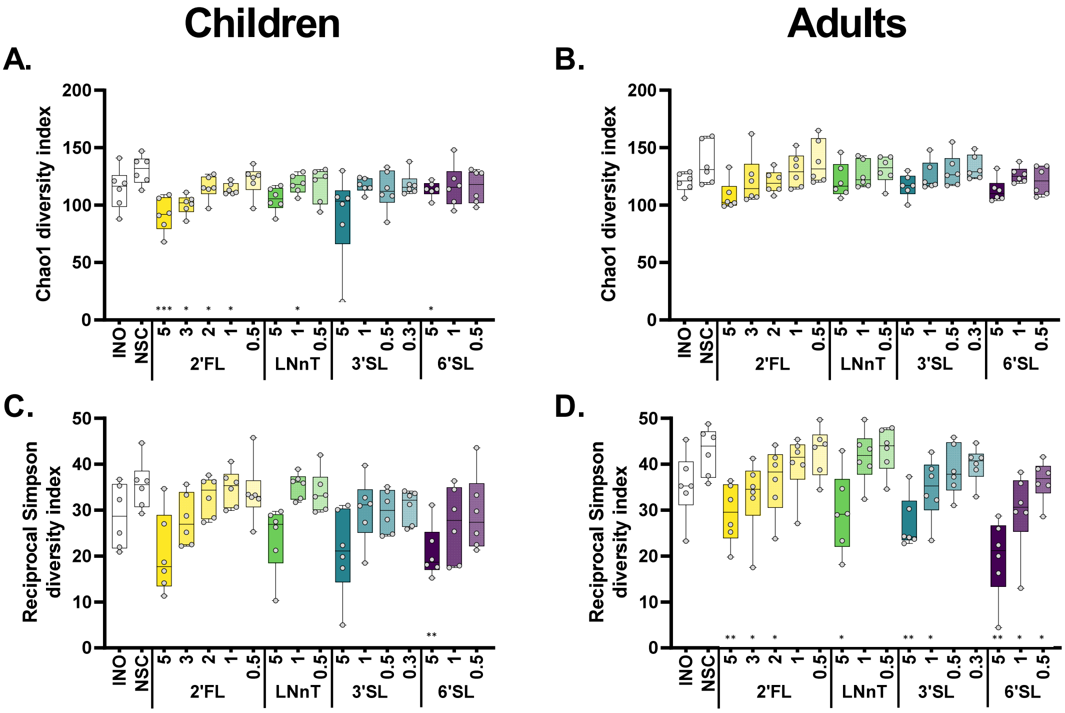

Figure S5. **When administered to the children's and adult microbiota, the four HMOs generally maintained a high microbial diversity.** Impact of four HMOs (2′FL, LNnT, 3′SL, 6′SL) at doses ranging from equivalent of 0.3 to 5 g/day on the Chao1 diversity index (A, B) and the reciprocal Simpson diversity index (C, D) for simulated gut microbiota of children (n = 6) or adults (n = 6), at 24h upon initiation of treatment, compared to a no substrate control (NSC), as tested with the *ex vivo* SIFR® technology. Statistical differences between treatments and NSC are indicated with asterisks [* ($p_{adjusted} < 0.05$), ** ($p_{adjusted} < 0.01$) or *** ($p_{adjusted} < 0.001$)].

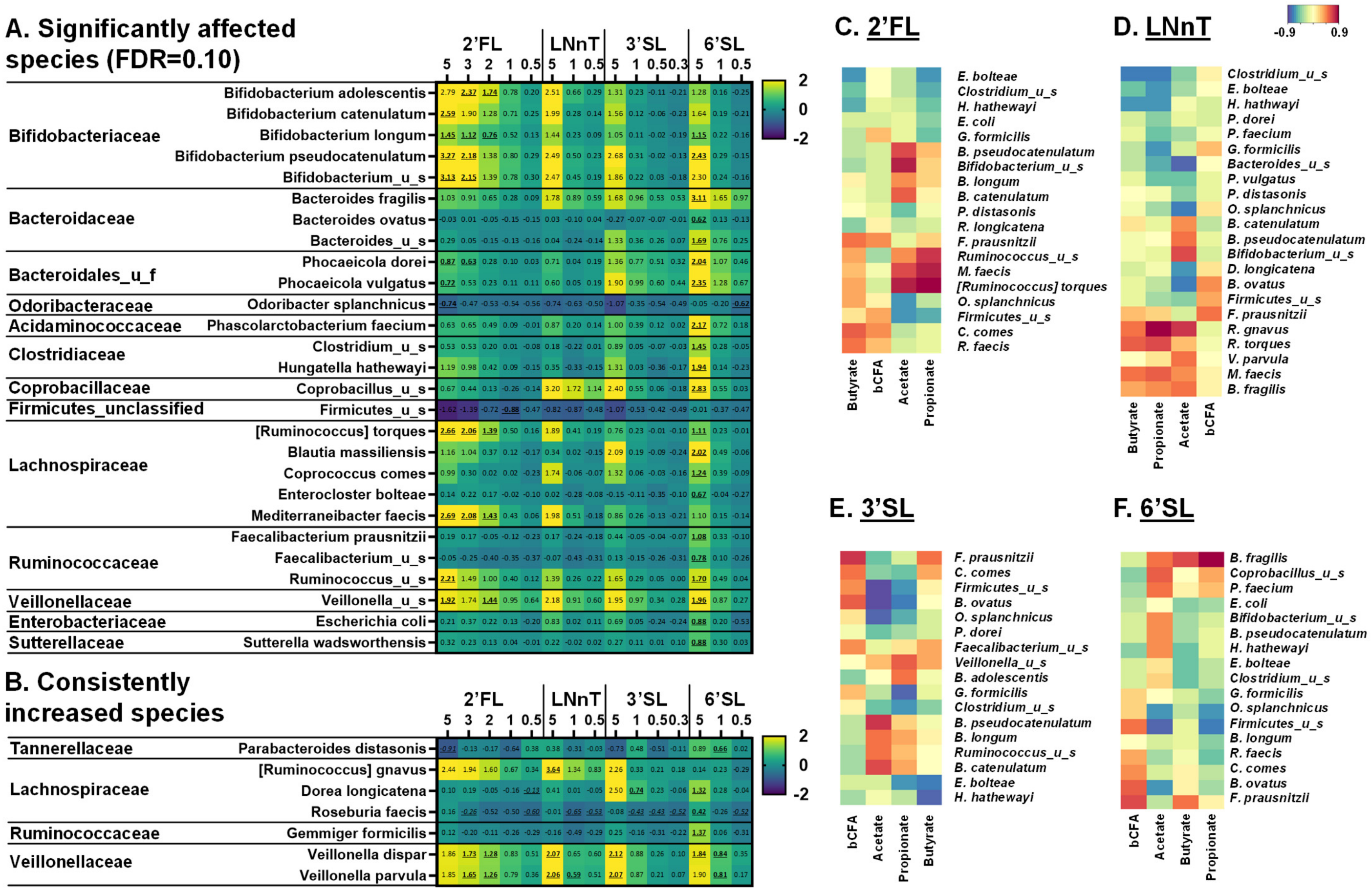

Figure S6. All HMOs impacted specific microbial species when supplied to the microbiota of children. Impact of four HMOs (2′FL, LNnT, 3′SL, 6′SL) at doses ranging from equivalent of 0.3 to 5 g/day on microbial composition at species level at 24h upon initiation of treatment, as tested with the *ex vivo* SIFR® technology for children. The heatmaps represent average values of microbial taxa that were (A) significantly (FDR = 0.10) (B) or non-significantly but consistently affected by any of the treatments (consistent = increased or decreased for all donors where they were detected). Significant/consistent differences are indicated by bold and underlining of the average log2 (abundance treatment/abundance NSC). (C-F) Regularized Canonical Correlation Analysis (rCCA) to highlight correlations between SCFA (acetate, propionate, butyrate), bCFA and significantly/consistently affected species, for each of the four HMOs.

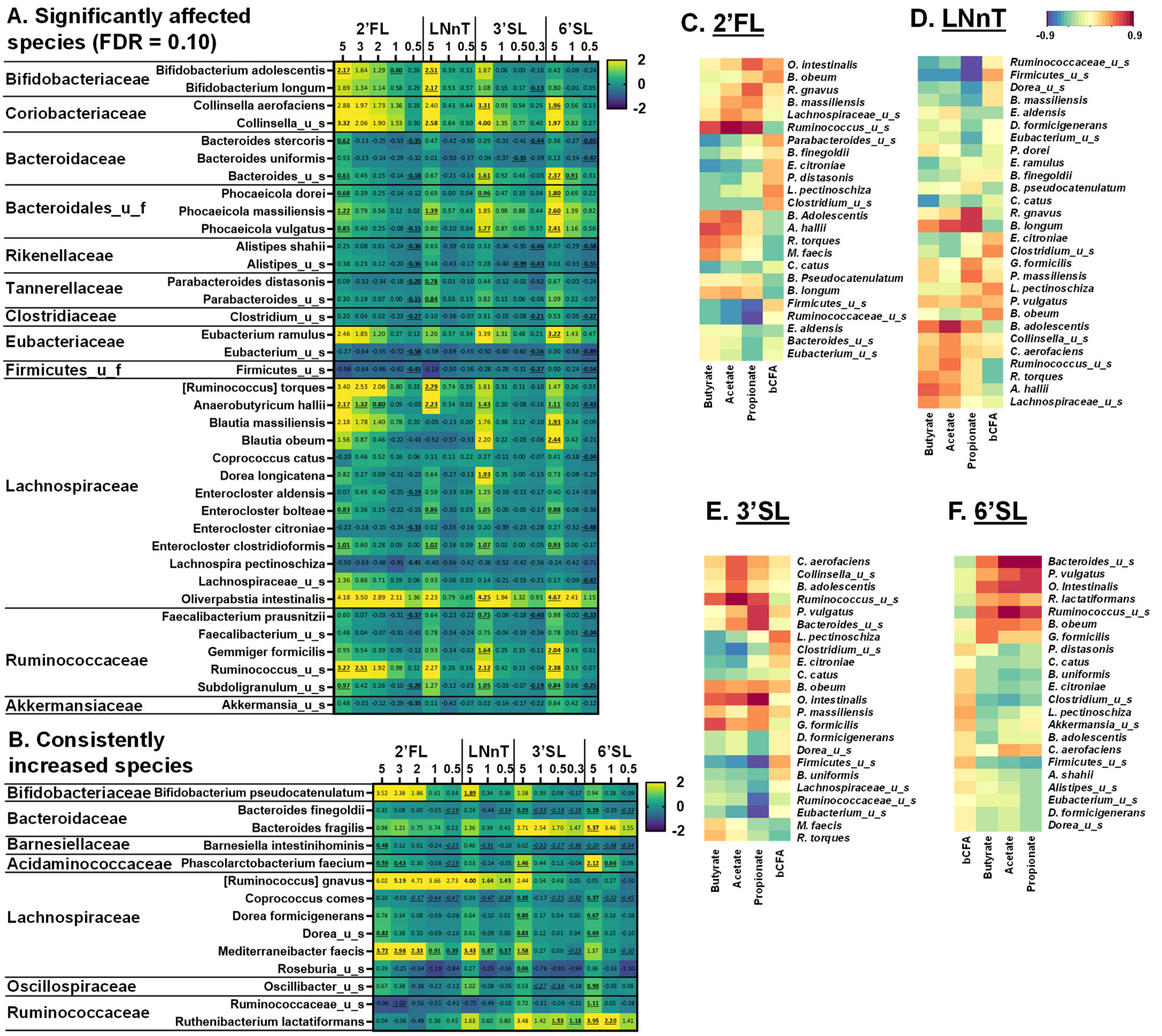

Figure S7. All HMOs impacted specific microbial species when supplied to the adult microbiota. Impact of four HMOs (2′FL, LNnT, 3′SL, 6′SL) at doses ranging from equivalent of 0.3 to 5 g/day on microbial composition at species level at 24h upon initiation of treatment, as tested with the *ex vivo* SIFR® technology for adults. The heatmaps represent average values of microbial taxa that were (A) significantly (FDR = 0.10) (B) or non-significantly but consistently affected by any of the treatments (consistent = increased or decreased for all donors where they were detected). Significant/consistent differences are indicated by bold and underlining of the average log2 (abundance treatment/abundance NSC). (C-F) Regularized Canonical Correlation Analysis (rCCA) to highlight correlations between SCFA (acetate, propionate, butyrate), bCFA and significantly/consistently affected species, for each of the four HMOs.

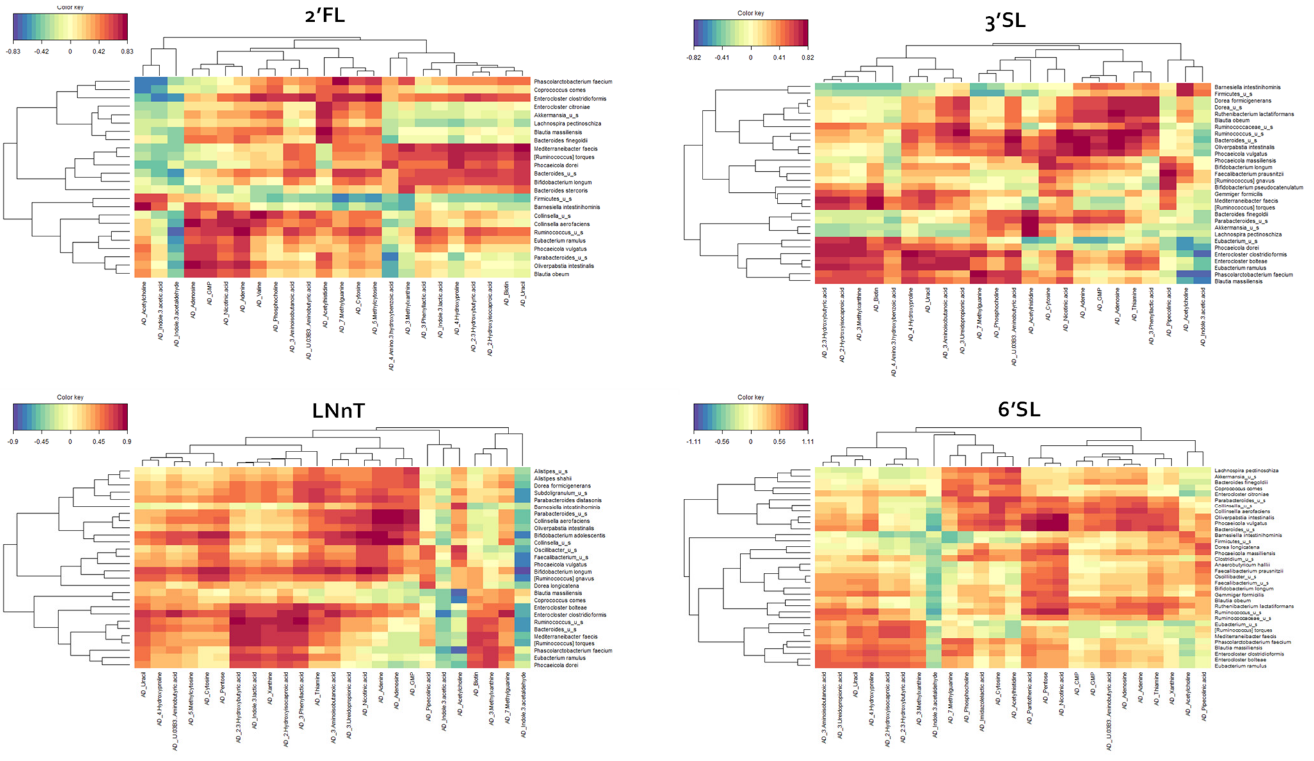

Figure S8. Regularized Canonical Correlation Analysis (rCCA) to highlight correlations between significantly affected metabolites and significantly/consistently affected species, for each of the four HMOs upon administration to the microbiota of children.

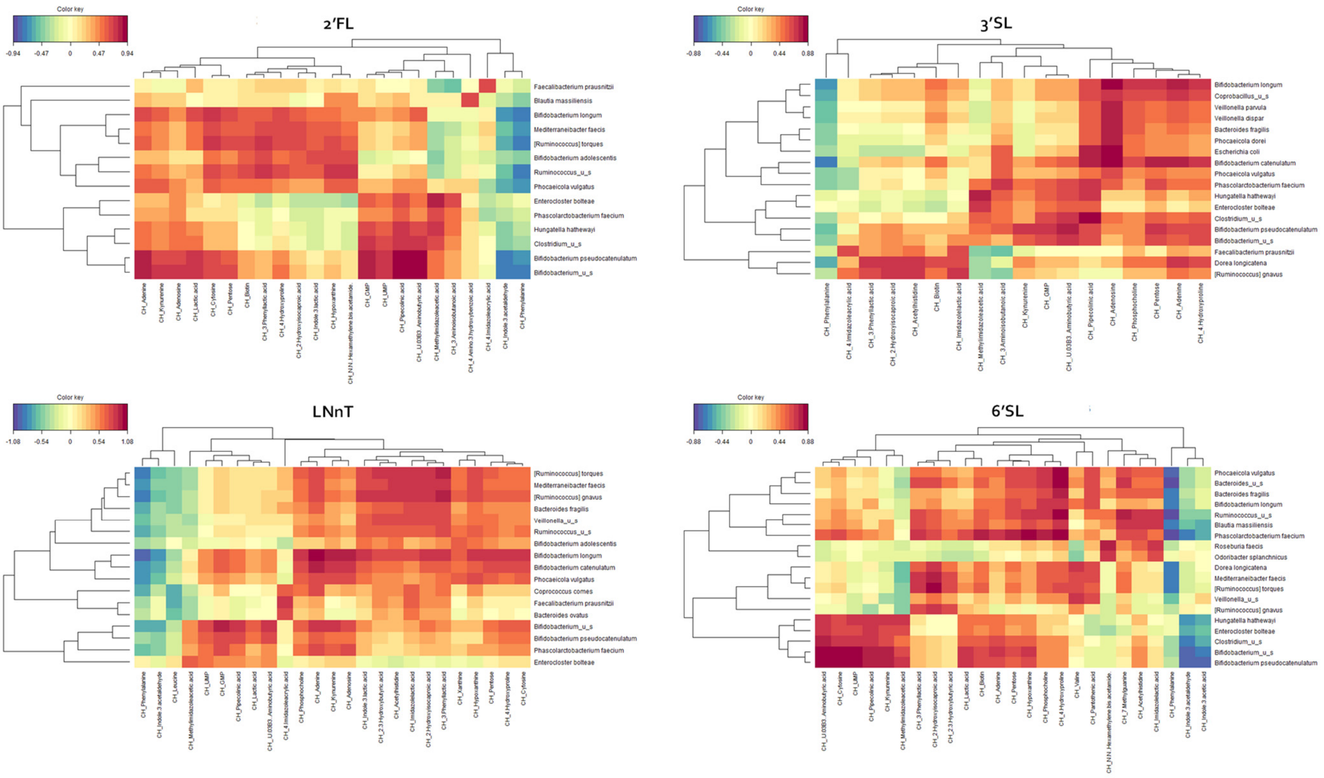

Figure S9. Regularized Canonical Correlation Analysis (rCCA) to highlight correlations between significantly affected metabolites and significantly/consistently affected species, for each of the four HMOs upon administration to the adult microbiota.
